# Appropriate CT Technology to Fully Assess a Palatal Myoepithelioma

**DOI:** 10.5334/jbsr.4168

**Published:** 2025-12-22

**Authors:** Xiang Zhuang, Hongliang Li, Qingyu Ji

**Affiliations:** 1 Baotou Cancer Hospital, Baotou, Inner Mongolia, China; 2 Second Affiliated Hospital of Baotou Medical College, Baotou, Inner Mongolia, China

**Keywords:** myoepithelioma, palatal mass, CT, hyper‑realistic rendering

## Abstract

*Teaching point:* Palatal myoepithelioma has almost pathognomonic progressive heterogeneous delayed enhancement on contrast CT, and a dedicated software application contributes to the surgical planning through hyper‑realistic rendering (HRR) 3D delineation of tumor extent/spatial relationships with critical adjacent structures.

## Case History

**Figure 1:**
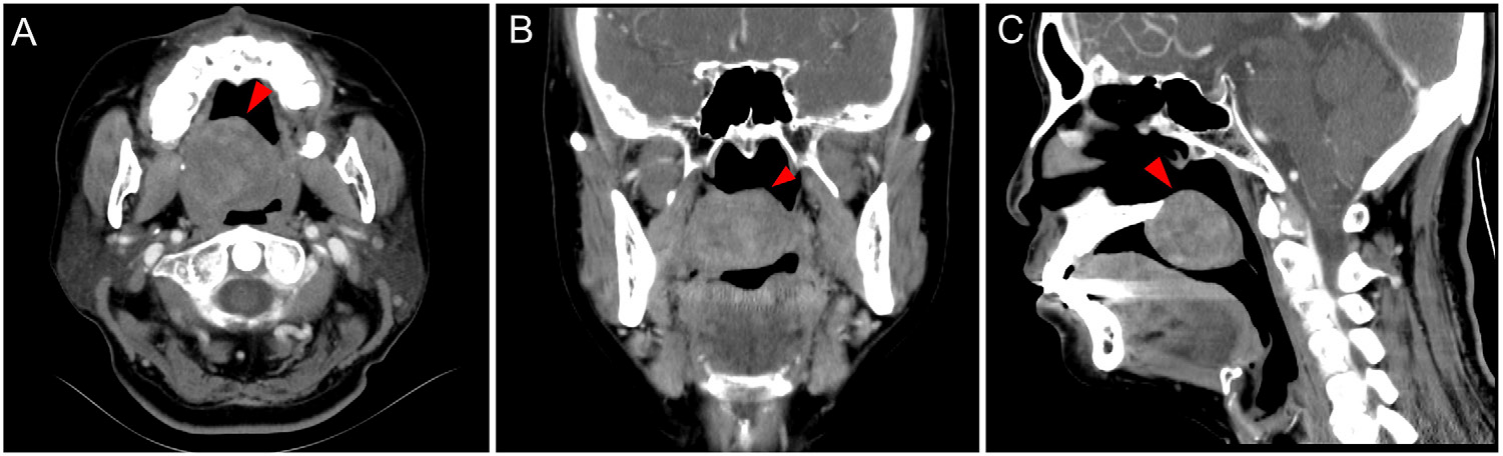
Enhanced CT images of the neck in a patient with palatal myoepithelioma.

**Figure 2:**
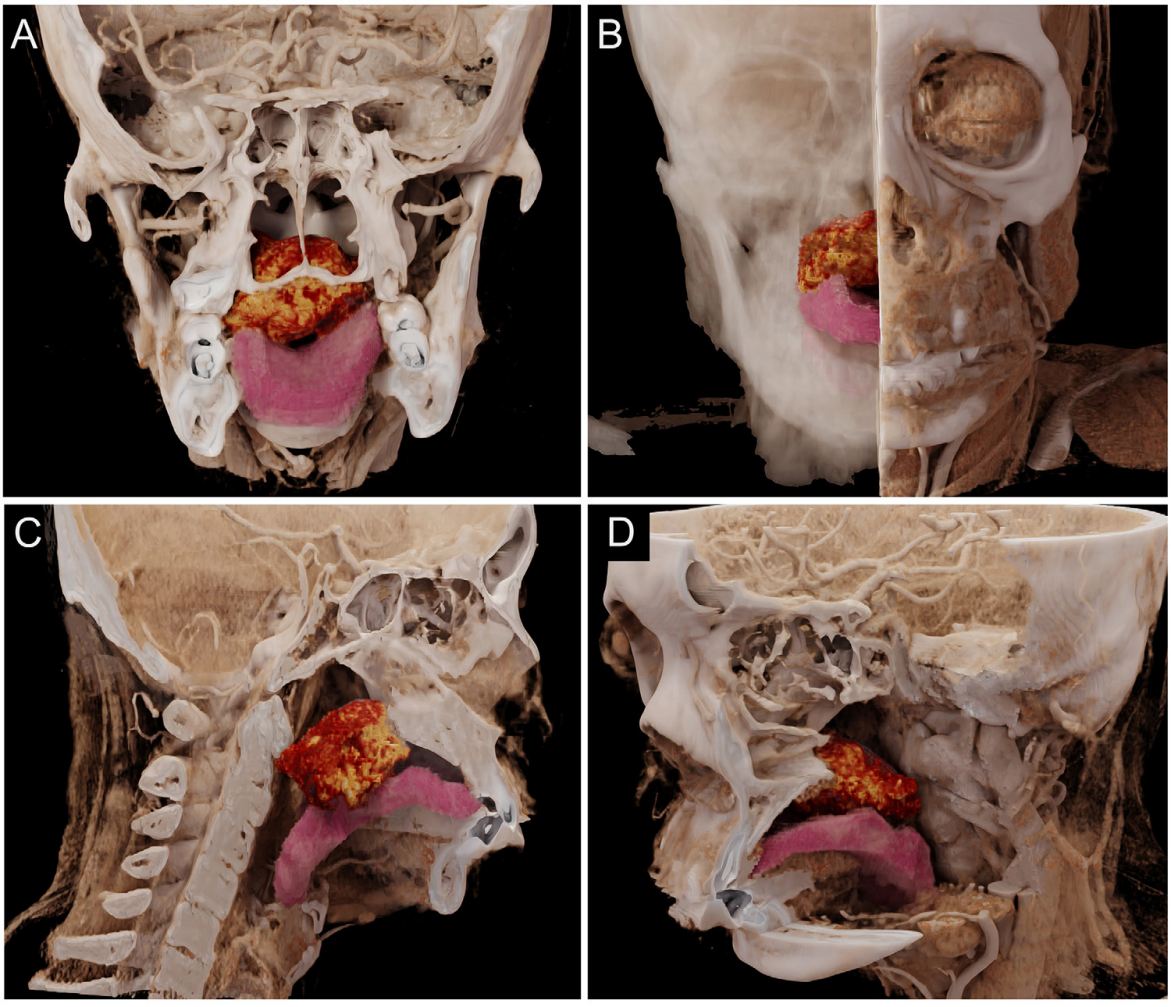
Hyper‑realistic rendering (HRR) of CT for palatal myoepithelioma.

A 46‑year‑old woman presented with a two‑year history of a painless palatal nodule, which she had initially disregarded due to the absence of symptoms. The lesion gradually enlarged, resulting in an oral foreign body sensation and difficult swallowing, which prompted her to seek medical attention. A contrast‑enhanced CT scan revealed an irregular, heterogeneously dense mass in the posterior palate, protruding into the oral cavity (Figure 1, red arrow). The tumor showed moderate heterogeneous contrast enhancement, with progressive delayed enhancement. The mass had ill‑defined borders with adjacent structures, including the right tongue base, uvular muscle, salpingopharyngeus, levator veli palatini, and tensor veli palatini muscles. No adjacent bone destruction was observed. For comprehensive presurgical assessment, CT imaging with hyper‑realistic rendering (HRR) was utilized (Figure 2). This technique clearly delineated the gross morphology, extent, and spatial relationships. The HRR images depicted the nodular mass in the posterior palate, revealing its irregular surface, the resultant narrowing of the oral cavity, and its spatial relationships with surrounding structures. Following this imaging‑guided assessment, the patient underwent surgical excision. Histopathological examination confirmed the diagnosis of myoepithelioma. Postoperatively, the patient’s symptoms resolved completely, and no recurrence was noted at the six‑month follow‑up.

## Comments

Myoepithelioma is a relatively uncommon benign tumor of the salivary glands, primarily composed of myoepithelial cells. It most frequently arises from the minor salivary glands of the palate and predominantly affects middle‑aged women [1]. The clinical presentation of ME is often non‑specific, typically appearing as a slow‑growing, painless mass that may be overlooked by patients, resulting in late diagnosis. Prolonged disease progression or recurrence carries a potential risk of malignant transformation. Contrast‑enhanced CT reveals progressive heterogeneous enhancement of the tumor, with hyperdense areas corresponding to vascular myoepithelial clusters and hypodense regions resulting from intratumoral collagenous or myxoid changes. Importantly, although ME is classified as a benign tumor, CT imaging in this case demonstrated obscured margins in relation to multiple adjacent muscular structures, such as the levator veli palatini and tensor veli palatini. This observation suggests that the tumor may exhibit more locally aggressive biological behavior or, at the very least, indicates close adhesion to surrounding tissues. Such findings are critical for preoperative surgical planning, underscoring the necessity for wide local excision.

CT‑based high‑resolution reconstruction offers significant advantages for preoperative planning through three‑dimensional, volumetric visualization of tumor morphology, extent, and spatial relationships with adjacent structures. This capability allows surgeons to more effectively identify critical anatomical landmarks, thereby improving the likelihood of complete tumor resection and minimizing intraoperative risks.

## Additional Files

The additional files for this article can be found as follows:

## References

[r1] Luccas GMG, Marinho KS, Cardoso LC, et al. Epithelial‑myoepithelial carcinoma: Retrospective analysis of the case series from a reference center in São Paulo, Brazil. Braz J Otorhinolaryngol. 2025;91(Suppl 1):101615. 10.1016/j.bjorl.2025.101615. 40446386 PMC12166795

